# The Implementation of Predictions During Sequencing

**DOI:** 10.3389/fncel.2019.00439

**Published:** 2019-10-09

**Authors:** M. Molinari, M. Masciullo

**Affiliations:** IRCCS Fondazione Santa Lucia, Rome, Italy

**Keywords:** sequencing, prediction error, forward internal model, cognition, emotions

## Abstract

Optimal control mechanisms require prediction capabilities. If one cannot predict the consequences of a motor act or behavior, one will continually collide with walls or become a social pariah. “Looking into the future” is thus one of the most important prerequisites for smooth movements and social interactions. To achieve this goal, the brain must constantly predict future events. This principle applies to all domains of information processing, including motor and cognitive control, as well as the development of decision-making skills, theory of mind, and virtually all cognitive processes. Sequencing is suggested to support the predictive capacity of the brain. To recognize that events are related, the brain must discover links among them in the spatiotemporal domain. To achieve this, the brain must often hold one event in working memory and compare it to a second one, and the characteristics of the two must be compared and correctly placed in space and time. Among the different brain structures involved in sequencing, the cerebellum has been proposed to have a central function. We have suggested that the operational mode of the cerebellum is based on “sequence detection” and that this process is crucial for prediction. Patterns of temporally or spatially structured events are conveyed to the cerebellum via the pontine nuclei and compared with actual ones conveyed through the climbing fibers olivary inputs. Through this interaction, data on previously encountered sequences can be obtained and used to generate internal models from which predictions can be made. This mechanism would allow the cerebellum not only to recognize sequences but also to detect sequence violations. Cerebellar pattern detection and prediction would thus be a means to allow feedforward control based on anticipation. We will argue that cerebellar sequencing allows implementation of prediction by setting the correct excitatory levels in defined brain areas to implement the adaptive response for a given pattern of stimuli that embeds sufficient information to be recognized as a previously encountered template. Here, we will discuss results from human and animal studies and correlate them with the present understanding of cerebellar function in cognition and behavior.

## Introduction

Literature data have shown that the brain is constantly making predictions about future events. Several theories of prediction in perception, action and learning suggest that the brain serves to reduce the discrepancies between expectation and actual experience, i.e., by reducing the prediction error (Brown and Brüne, 2012).

Predictive ability may indeed map well to Prefrontal cortex (PFC) in addition to primary sensory areas, with significant portions of PFC specialized for reporting error as a deviation from predicted events (Alexander and Brown, 2018).

The idea that also the cerebellum is involved in predicting the effects of motor commands is well accepted in the neuroscience community (Bastian and Thach, 2002; Popa et al., 2012; D’Angelo and Casali, 2013; Pisotta and Molinari, 2014). The role of the cerebellum in cognition and emotion remains more heavily debated (Koziol et al., 2014), although it is almost generally accepted that the cerebellar structures are involved in cognition.

In the framework of cerebellar cognition, different studies, research groups and cerebellar clinical centers have provided sample data demonstrating cerebellar output to the cerebral cortex as the cornerstone for understanding basic cerebellar functioning (Molinari et al., 2005; Timmann et al., 2010).

Integration of cognitive and motor cerebellar functions forced a reconsideration of the basic operational mode of the cerebellum, and among the theories on cerebellar functioning (for a recent review on cerebellar theories see D’Angelo and Casali, 2013), sequencing has been considered suitable for describing cerebellar cognitive processing (Molinari et al., 2005).

In this context, sequence processing was suggested as the basic functional mechanism of the motor (Braitenberg et al., 1997) and cognitive (Molinari et al., 1997, 2009; Molinari, 2016) functions.

Sequencing has been defined as “the ability to perceive, represent and execute a set of actions (events) that follow a particular order” (Savalia et al., 2016). This is a sovramodal function present in virtually all human activities and even in many processes at neuronal level. According to this definition, sequencing can be recognized in the cellular capacity to detect a spike sequence as well as in recognizing a given firing in a neuronal network.

Eye Blink Classical conditioning can be considered the simplest unitary component of sequence planning and it represents one of the more productive area of cerebellar research; moreover, literature data from different groups provided evidence of sequence processing mechanisms at circuitry and cellular level (Bracha et al., 2009; Swain et al., 2011).

In this ability, the cerebellum with its peculiar anatomical organization is well equipped for paying a central role. As proposed by Braitenberg et al. (1997), cerebellar capacity to tag time and space characteristics of inputs is embedded in the cortico-nuclear microcomplex structure (D’Angelo and Casali, 2013). Signals traveling through the parallel fibers possess precise spatio-temporal features. These in turn determine the specificity of the cerebellar nuclei output. “What the beam passes on to the cerebellar nuclei is a sequence of signals produced by selected Purkinje cells at times specified by the moving wave of excitation.” Particularly in the sensory domain, different experimental models were instrumental in depicting theories on cellular mechanisms for prediction of sensory events (Mauk and Ohyama, 2004; D’Angelo and Casali, 2013; Yamazaki and Lennon, 2019).

The hypothesis that sequence detection might represent the main contribution of cerebellar physiology to brain functioning is presented and discussed here.

## Cortico-Cerebellar Crosstalk

The history of research into the connections between the cerebellum and the cerebral cortex is quite long, and many aspects still await clarification. Cerebellar terminals in the thalamus were described in non-human primates in the early 1980s (Asanuma et al., 1983) and corresponding areas were revealed in humans more than a decade later (Macchi and Jones, 1997). A clear step forward in experimental tract-tracing studies derived from the use of transneuronal transport of viruses. Experiments in primates indicated that the motor, premotor, prefrontal and parietal cortices receive cerebellar information via the thalamus (Strick et al., 2009). Functional connectivity magnetic resonance imaging studies confirmed widespread cortico-cerebellar interconnections well beyond motor areas (Allen et al., 2005; Palesi et al., 2017).

The cortico-cerebello-cortical loop is believed to be organized in parallel segregated modules (Ramnani, 2006). If this is true, then cerebello-cortical functional interactions can be quite specific and can be dynamically organized in continuously changing patterns allowing specific crosstalk between the cerebellum and cortex to meet the ever-changing requests needed to optimize brain activity.

Despite the well-advanced characterization of cerebro-cerebellar organization, its function remains poorly understood. Neurophysiological techniques, in healthy subjects and in patients, have been instrumental in clarifying interactions between the cerebral cortex and cerebellum (Tesche and Karhu, 1997, 2000; Ivry, 2000; Nixon, 2003; Molinari et al., 2005).

Thus, the quest to identify the cerebellar processes underlying the modulation of cortical activity is well under way. One of the main intriguing aspects, as noted by many since early times, is the apparent contradiction that cerebellar circuits organized in a uniform structure but involved in many different functions. Different theories have been put forward to identify the basic operational mode of the cerebellum and thus decode its influence on so many functional domains. Error detection (Marr, 1969; Albus, 1971; Ito, 1990), timing (Ivry and Keele, 1989), sensory processing (Bower and Parsons, 2003), and sequence detection (Braitenberg et al., 1997) are among the most widely accepted theories. In particular, the sequence detection hypothesis is advanced to highlight the peculiar role of the cerebellum in the functional organization of the predictive brain network. On the other hand, the same hypothesis has been proposed as the basic operation mode of the cerebellum in all the multifarious domains reported to be affected in patients with cerebellar damage. In summary, the sequence detection theory postulates that the cerebellum is capable of detecting and memorizing patterns, constructing internal models of the perceived patterns.

**FIGURE 1:**
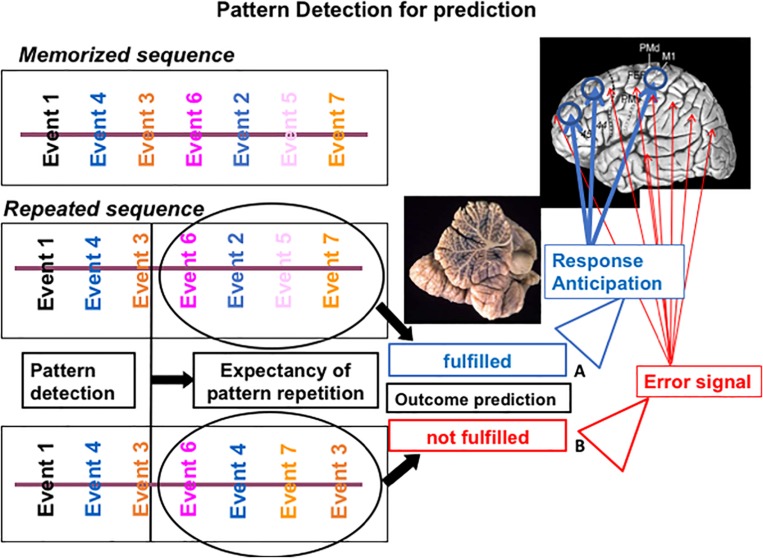
Putative mechanism of cerebellar sequencing for prediction incoming events are continuously monitored in the cerebellar circuits. Relations between events are compared in the cerebellar circuits (Ito, 2006) and stored in a working memory area. When the sequences of new incoming events occur, they are compared with previously stored event ones. If a match is recognized **(A)**, then an expectancy of repetition is generated and the feedforward control can function efficiently. If prediction fails **(B)**, then an error signal is activated by the cerebellar output system, and feedforward control is interrupted or corrected.

If an activity pattern resembles a memorized pattern, then precise expectations linked to the identified internal model are activated. The correctness of the prediction is estimated by confronting bottom-up incoming information with top-down expectations. If the prediction holds, the specific brain areas previously successfully used to respond to that stimulation pattern are selectively activated, thus allowing a more efficient response. Conversely, violation of expectancy will induce general brain activation and thus a less efficient response (see Figure 1).

A study involving a large population with focal or degenerative cerebellar pathologies reported sequencing to be the most affected cognitive domain (Tedesco et al., 2011).

Interestingly, sequencing has been shown to be relevant for understanding the cerebellar role in pathophysiological mechanisms in different conditions, such as schizophrenia (Shergill et al., 2014) and autism (Larson and Mostofsky, 2008), in which impairments in patterns of information processing and disruptions in error signal prediction have been proposed.

## Cerebellum and Its Role in Predicting Perception

To achieve mind-world synchronization, our perceptual systems must constantly tune themselves to an ever-changing environment.

“Looking into the future” is one of the most significant concepts in neuroscience (Bubic et al., 2010). As recently argued by Pisotta and Molinari (2016), the brain is constantly required to predict future events. This process is critical for many aspects of information, such as perception, motor and cognitive control, decision-making, and theory of mind, to name just a few.

One of the main abilities allowing the brain to adapt to a changing environment is the capacity to correct errors. Within this framework, looking into the future represents the best way to avoid errors. Among the areas constituting the “predictive brain,” the cerebellum and its ability to generate internal models are hypothesized to play a central role.

As elegantly stated by Ebner, 2013 (Cerebellum and Internal Models, Handbook of the Cerebellum and Cerebellar Disorders, 2013), “There are two general classes of internal models. Forward models use the commands for an action and information about the present state to predict the consequences of that action. Inverse models transform a desired outcome or effector state into the necessary commands to achieve that state.”

We recently synthetized Ebner’s theory, depicting two conditions: “(1) the cerebellum provides the motor system with the correct sensory information that is needed to adjust movements in real time, or (2) the cerebellum identifies sensorimotor patterns that fit into known motor sequences and thus can prepare the cortex for the next step. The first hypothesis postulates that cerebellar activity is related to ongoing motor or sensory information. In the second, cerebellar activity is related more to the expectancy of future events than with the registration of ongoing activities” (Molinari et al., 2009).

Ebner and Pasalar, 2008 argued that “the spike discharge of monkey Purkinje cells does not have the dynamics-related signals required to be the output of an inverse dynamics model signals.”

On the other hand, the neurophysiological data are more in line with the idea of a forward internal model. Overall, the cerebral cortex receives information on future events from Purkinje cell firing. Through this mechanism, the cortical modules needed to respond to the foreseen condition will be alerted in advance.

It must be noted that the preparatory function of the cerebellum cannot be limited to a single functional domain. Overall, the capacity of the cerebellum to predict incoming inputs (Tesche and Karhu, 2000), and thus alert specific brain circuits (Restuccia et al., 2007; Moberget et al., 2008) can be considered a supramodal function. Consequently, prediction capability affects whole-brain function, alerting the specific neural systems (e.g., sensory, motor, autonomic, memory, attention, affective, speech, and language) required to respond to a given context.

Tesche and Karhu (2000) analyzed the neural signals generated in the somatosensory cortex and cerebellum according to the predictability of a sensory stimulus. When the stimulus is absent, no activity is present in S1, as expected, whereas the cerebellar response is evident and is much larger than the one recorded when the stimulus is present. The most direct interpretation indicates that the cerebellum reacts to the absence of an expected somatosensory stimulus more than its presence. This response to the absence of a stimulus can be understood only as an indication that something that is expected does not appear (Ivry, 2000). When sensory patterns are recognized, prediction of sequence of events is possible, and consequently, the appropriate brain state can be established beforehand (Nixon, 2003). What is the content of such prediction? Somatosensory Evoked Potentials (SEPs) are presented in a fixed time frame; thus, cerebellar activity may signal the absence of an expected sensation as well as a deviation from expected timing (Ivry, 2000).

The theoretical framework to reconcile the two views is sequencing. By definition, relationships in time and space are the building element of a sequence (Molinari, 2016).

To test the role of the cerebellum in prediction vs. timing, Restuccia et al. (2007) adopted a somatosensory mismatch negativity (MMN) paradigm in which oddballs were generated by varying not the rhythm but the location of the stimuli (Restuccia et al., 2007). Oddball signals were generated by interspersing fifth-finger stimulation among frequent left-thumb stimulations. This s-MMN paradigm was studied in subjects with unilateral cerebellar lesions to exploit the possibility of testing cortical responses with and without cerebellar processing in the same subject. Because of the well-known crossed organization of cerebro-cerebellar circuits, unilateral cerebellar damage will affect only the cerebral cortex of the contralateral hemisphere (Di Lazzaro et al., 1994a, b, 1995; De Vico Fallani et al., 2016).

As we already argued in 2008 (Molinari et al., 2008), considering the involvement of the cerebellum in the prediction of sensory events (Nixon, 2003) and the old theory that it acts as a comparator (Ito, 2006), it is plausible that actual inputs and preceding stimuli are compared within the cerebellum and discordances are tested. If the incoming stimulus matches the predicted stimulus, cerebellar output is not significant; if a discrepancy–error signal is identified, then the output of the cerebellum increases, and a large area of the cerebral cortex is alerted by increasing its excitability.”

## Prediction in Locomotion

Locomotion is a complex act that involves, in addition to basic locomotor motor patterns provided by spinal interneuronal networks (CPGs), different control centers, both in subcortical and cortical areas (Takakusaki, 2013), including the cerebellum.

The role of the cerebellum in locomotor control and learning has been demonstrated in animals by electrophysiological studies. The spinocerebellum is one of the main structure that processes information conveyed by peripheral sensory signals and information from the spinal pattern generators through the spinocerebellar tracts (Arshavsky et al., 1983; Fedirchuk et al., 2013). Recordings of spinocerebellar neural activity revealed that step-related information is present in the activity of many cerebellar neuron types. An essential role for interlimb coordination, adaptation to external perturbation, is played by Purkinje cells, which tend to fire rhythmically with the stepping cycle (Udo et al., 1981; Armstrong and Edgley, 1984; Yanagihara and Kondo, 1996).

How the cerebellum normally contributes to locomotor behavior in humans is debated, although recent works suggest that it helps generate appropriate patterns of limb movement, dynamically regulate upright posture and balance, and adjust the feedforward control of locomotor output through error-feedback learning.

The role of the cerebellum in the timing and scaling of individual joint movements during gait was addressed by Earhart and Bastian (2001) (J Neurophysiol). The authors asked individuals with cerebellar lesions to step on an inclined surface while walking.

Based on the changes in inclination, healthy subjects presented systematic shifts in the timing of muscle activity and peak joint angles, thus mastering the task through several temporal strategies. Notably, subjects with cerebellar lesions presented appropriate timing shifts at most joints, thus demonstrating preservation of the basic timing of motor patterns. Conversely, relative joint movements were abnormal with movement decomposition, implicating the cerebellum in multiple joint adjustments, particularly when external constraints must be accommodated (Earhart and Bastian, 2001). With the sequencing theory in mind, it appears conceivable that, in presence of cerebellar damage, motor timing is preserved, while multi-joint coordination, requiring spatio-temporal sequence processing, is not.

At present, clinical and experimental data support the idea that cerebellum processes information for adaptive gait control, allowing constant recalibration of walking patterns to smoothly adapt to various terrains and environments. Subjects affected by cerebellar damage are impaired in locomotor tasks that require prediction, whereas they have good control when reactive control is needed (Morton and Bastian, 2006). This evidence demonstrates that cerebellar adaptation is based not on sensory feedback information but on prediction.

Moreover, several studies investigated the biomechanical characteristics of patients with degenerative cerebellar atrophy (spinocerebellar ataxia, or SCA), finding these to consist of decreases in step length, gait speed, and ankle torque; increased step width; impaired interjoint coordination; and marked variability of all global segmental gait parameter values (Palliyath et al., 1998; Mitoma et al., 2000; Earhart and Bastian, 2001; Morton and Bastian, 2003; Serrao et al., 2012; Wuehr et al., 2013). Moreover, previous findings (Konczak and Timmann, 2007; Bastian, 2011; Goodworth and Peterka, 2012; Timmann et al., 2013) suggest that lesions of the cerebellum may induce abnormalities in the spatial and temporal pattern of muscle activation resulting in specification gait impairments. In this regard, Martino et al., 2014 (J Neurophysiol 2014) found that SCA patients showed a widening of muscle activation profiles as a consequence of improper motor planning (feedforward control) and processing of proprioceptive information (Bastian, 2011), leading to inaccurate movements.

Sequencing intervenes at various levels of locomotor control, providing the basic mechanism for sustaining prediction. As observed in sMMN paradigms (Restuccia et al., 2007), it can be argued that, during locomotion, the cerebellum recognizes fixed sequences of sensory information (Pisotta and Molinari, 2016) funneled by spinocerebellar fibers (Jankowska et al., 2011). Through this mechanism, a correct prediction of the neuromuscular requirements of the subsequent step is achieved. If the actual sequence does not match the predictive sequence, then the cerebellar output system will be enhanced, allowing cortical and brainstem locomotor regions to adapt.

In other words, advance information on subsequent step events (feedforward control) is achieved through cerebellar sequencing, further supporting the idea that sequencing is the basic operational mode of the cerebellum. Recent data in mice provide support to this hypothesis (Darmohray et al., 2019). Chemogenetic dissection of cerebellar circuitries using a split belt locomotion learning paradigm, indicated that spatial and temporal components of gait are both encoded by Purkinje cells (Darmohray et al., 2019). This evidence indicates that timing is not the only domain in which cerebellar control is exerted, indicating spatio-temporal sequencing the best candidate of basic cerebellar operational mode.

## Prediction in Cognition

Since the last century, the ideas on cerebellar functioning have been completely transformed. Even in the 1990s, neurophysiology text books were still presenting an oversimplified functional view of cerebellar functioning with all cerebellar competencies restricted to the motor system. Currently, cerebellar circuits are identified as part of most brain networks, thus indicating involvement not only in motor control but also in virtually all aspects of cognition.

Notwithstanding early reports since Luciani’s work (Manni and Petrosini, 1997), a consensus on the cognitive function of the cerebellum was only recently formed.

Anatomical and neuroimaging investigations on cortical-cerebellar connections provide the neurobiological basis for the cerebellar contribution to cognitive functions. Functional MRI studies revealed activation of the cerebellum during several cognitive tasks, particularly in experiments that employed working memory or executive functions (Durisko and Fiez, 2010; Marvel and Desmond, 2010; Chen et al., 2014; Castellazzi et al., 2018).

Cerebellar activation is not limited to this modality but is also present in tasks involving attention and timing (Akshoomoff and Courchesne, 1992; Xu et al., 2006). Regarding language, studies indicate prominent activation of the lateral cerebellar hemispheres (Stoodley and Schmahmann, 2009).

In addition to neuroimaging data, data from preclinical models and clinical studies document diverse cognitive deficits associated with cerebellar damage. The list includes impairments in executive function, procedural memory, declarative memory, and associative memory tasks such as eye blink conditioning, along with deficits in timing/attention (Schmahmann and Sherman, 1997, 1998; Ravizza et al., 2006; Gerwig et al., 2008; Koziol et al., 2014; Baumann et al., 2015).

Recently, in the context of an experimental work on the role of the cerebellum in a countermanding task, we had the opportunity to summarize our view defining the role of the cerebellum in error control across domains (Olivito et al., 2017). One prominent postulation concerning cerebellar involvement in non-motor domains is based on the idea that the cerebellum allows online prediction of upcoming occurrences and produces estimates of future states by implementing internal models (see Figure 1). This mechanism allows the system to anticipate predictable events and consequently modify behavior when these predictions are violated (Ivry and Spencer, 2004; Ghajar and Ivry, 2009; Molinari et al., 2009; Leggio and Molinari, 2015; Moberget and Ivry, 2016).

For example, several studies revealed that the cerebellum contributes to the decoding of errors and to the consequent behavioral adaptation in both cognitive and motor domains (Blakemore et al., 2001; Molinari et al., 2008, 2009).

In the results of Ide and Li (2011), the cerebellum emerges as an important structure strongly modulated after error experience in the countermanding task, in cooperation with the ventrolateral PFC and the thalamus (Li et al., 2008). Furthermore, specific impairments in subjects with focal or atrophic cerebellar damage have also been reported (Brunamonti et al., 2014; Olivito et al., 2017). Thus, together with the PFC, anterior cingulate cortices, basal ganglia, and supplementary motor areas, the cerebellum is part of a distributed network contributing to the elaboration of errors as “deviations from what is expected” and to performance monitoring in general (Chevrier and Schachar, 2010; Peterburs et al., 2015).

A previous work documented that subjects with cerebellar damage developed impairments in cognitive sequencing (Leggio et al., 2008). Leggio et al. (2008) using a card-sequencing test, analyzed the ability of patients affected by cerebellar lesions to reconstruct the correct sequence of a set of cards, specifically differentiated with regard to the material (verbal, spatial, or behavioral) that was to be sequenced (Leggio et al., 2008). The patients presented with clear cognitive sequencing impairments independent of the material that was to be processed.

Consequently, the authors stated that the cerebellum identifies serial events as a sequence, finds a sequence violation, and is able to reconstruct the correct sequence of events. The hypothesis that pattern detection, prediction and processing of anticipation are cerebellum-dependent functions is similar to the sequence detection hypothesis in that it links the multifarious impairments that are reported in patients affected by cerebellar damage (Leggio et al., 2008; Molinari and Leggio, 2013).

## Prediction in Behavior

Behavior control relies on a complex network, and recently, cerebellar circuits have been considered relevant. Examining early reports, it has been observed since the 1800s that deviant and aberrant behaviors are present in patients affected by cerebellar anomalies (Schmahmann, 1991). Subsequent clinical studies (Cooper and Upton, 1978) reported a correlation between psychosis and cerebellar damage.

Schmahmann and Sherman (1998), in their initial description of cerebellar cognitive affective syndrome (CCAS), described significant behavioral disruption in 20 patients with cerebellar damage, with behavioral manifestations ranging from affective changes to behavioral disinhibition.

Several authors (Bower et al., 1981; Schmahmann and Sherman, 1998; Andreasen and Pierson, 2008) suggested that the cerebellum regulates mental operations in much the same way as it regulates movements.

The psychiatric literature provides many interesting data highlighting the role of the cerebellum in behavioral control, particularly in schizophrenia. Within the framework of cerebellar involvement in schizophrenia, the connections and cellular architecture of the cerebellum support an interesting theory explaining the different symptoms of this pathology. It is not conceivable that the only dysfunctional brain structure in schizophrenia is the cerebellum. Rather, schizophrenia is probably a disease involving the interaction between multiple components in distributed brain circuits. If this is true, then no structure is necessarily the primary pathological site. Conversely, the network-based theory implies that on any given occasion, or during any given task, different nodes of the network may malfunction in a way that affects the whole system. Alternatively, malfunction might be derived from altered interactions among nodes of the distributed circuits (e.g., cortical areas, thalamus, and cerebellum).

Clinical and experimental findings indicate that schizophrenic patients estimate time less accurately than healthy controls do (Giersch et al., 2016). Schizophrenia is associated with attention deficits and working-memory impairment (Cohen et al., 1997). Moreover, patients affected by schizophrenia can remember that an event occurred but do not know when it occurred. These data have been interpreted considering that patients do not lose memory but that time perception is disorganized (Capa et al., 2014). Overall, many researchers have indicated that time perception is impaired in schizophrenia (Cohen et al., 1997; Giersch et al., 2016).

On the other hand, it has been proposed that psychotic symptoms depend on the lack of coherence between internally perceived and externally generated signals (D’Angelo and Casali, 2013). This “mind-world synchronization” can be obtained when perceptual mechanisms are constantly tuned to an ever-changing environment (Paquette et al., 2013); thus, perceptual tuning is achieved when patterns are recognized and predictions fulfilled (Molinari et al., 2008).

As proposed by Braitenberg et al. (1997), we applied a “sequence detection model” to highlight the cerebellar operational mode in several domains, including the processing of emotions (Molinari et al., 2008; Lupo et al., 2015; Adamaszek et al., 2017; Clausi et al., 2018).

This theory states that the role of the cerebellum in proactive and flexible control of behavior (Miall, 1998; Schlerf et al., 2012) is achieved by implementing a forward model of the incoming sensory input (Wolpert and Kawato, 1998), in turn affecting the cortico-subcortical network involved in error processing and corrective behavior (Falkenstein et al., 2000; Ullsperger and von Cramon, 2006).

Starting from observations in subjects with alterations of cerebellar circuits because of atrophy, we hypothesized that cerebro-cerebellar interactions are altered through continuous error signaling due to misdetection of incoming sequencing. This will induce insertion of virtual errors in the forward control models, thus generating continuous correction of the ongoing motor program (Pisotta and Molinari, 2016).

This hypothesis, derived from observations in the motor system, could help in understanding schizophrenia symptoms. In this latter condition, it can be argued that an incorrect error signal could misguide a sequence/pattern of behavior during the adaptation of behavior to context.

Overall, at the behavioral level, despite the organization based on the function-specific input and networks, the cerebellum plays a unique role in acquiring and predicting sequences affecting not only the understanding of planned and observed actions but also the construction of internal mental models. The role of the cerebellum in this function would be more demanding when applied to novel or complex sequences. These hypotheses are admittedly still at an early stage.

## Conclusion

Sequencing refers to the ability to acquire knowledge of the structure of sequences. This can be achieved incidentally acting on event sequences through experience or, in case of explicit efforts, intentionally. To learn a sequence means that the presentation and ordering rules of stimuli must be acquired. The working memory system comes into play by keeping the information on a single stimulus active, allowing comparison with subsequent stimuli. In addition, the relationships among the temporal and spatial characteristics of the stimuli must be acquired. Of relevance is the need to store the sequence structure once identified.

Sequencing is not recognized as a discrete cognitive function. Sequencing abilities are relevant in various fields of cognitive neuroscience. The network subserving sequencing involves different regions, and functional hypotheses have been advanced. For example, predictive functions have been suggested for frontal regions (Bubic et al., 2010), spatial sequencing processing in the hippocampus (Iglói et al., 2010; Babayan et al., 2017) and spatial-temporal relationships in the cerebellum (Leggio et al., 2011; Molinari, 2016; Babayan et al., 2017). Overall, the relationship between sequencing and other functions such as working memory and timing is still elusive, and we can consider it supramodal. In line with this hypothesis, deficits in sequencing affects many discrete domains, and compensation is quite effective.

Within the framework reviewed in the previous paragraphs, sequencing and the cerebellum appear to be closely linked. Regardless of the material processed, comparisons among actual and preceding patterns, as well as detection of discrepancies, occur in the cerebellum (Molinari et al., 2009).

Data from animal and clinical studies converge in supporting this view of fundamental cerebellar operation. Nevertheless, it is still not clear whether cerebellar comparison focuses mainly on time as suggested by Ivry (2000) or integrates processing of spatial and temporal characteristics (Leggio et al., 2011; Darmohray et al., 2019). Another relevant aspect is where internal models coded as pattern/sequence information are stored. The cerebral cortex, basal ganglia and cerebellum are all likely candidates (Leggio et al., 2011). Similarly, experimental and clinical evidence are prompting cerebellar function models to take sequencing in consideration (Tanaka et al., 2019; D’Angelo and Casali, 2013; Molinari et al., 2018; Rowan et al., 2018). Further studies should address the specific roles of these structures in sequencing, particularly to better understand predictive brain mechanisms.

## Author Contributions

Both authors wrote the manuscript.

## Conflict of Interest

The authors declare that the research was conducted in the absence of any commercial or financial relationships that could be construed as a potential conflict of interest.
